# College of Paramedics Research Conference 2024

**DOI:** 10.29045/14784726.2024.9.9.2.44

**Published:** 2024-09-01

## Dragon’s den

### Can pre-hospital clinicians reliably rule out raised intracranial pressure using optic nerve sheath diameter measurements with point-of-care ultrasonography in adult head trauma patients without clinically significant signs and symptoms?

**Benjamin Mudd**

University of East Anglia Email: benjamin.mudd@eastamb.nhs.uk

#### Abstract

Head trauma, or suspected head trauma, makes for a large percentage of pre-hospital attendances and subsequent admissions, based on current NICE guidelines, especially where anticoagulant therapy is in use with adult patients.

My proposal will lay out the data supporting the need for a PICO comparison study, whereby measurements of a patient’s optic nerve sheath diameter (ONSD) are taken prior to admission and then compared to CT head findings in order to demonstrate that the correlation between ONSD findings can effectively rule out the need for admission for CT head.

I would enrol a selected number of pre-hospital advanced clinical practitioners across ambulance service trusts to ensure there is high heterogeneity across similar systems and a variety of patient demographics for all adult patients with known or suspected head trauma and without signs and symptoms that would exclude them from the study. We would either enrol the patient at the time of attendance and gain consent from them or gain access to CT head findings via the pre-existing pre-hospital emergency medicine feedback process in the selected hospital trusts.

We would use an agreed point-of-care ultrasonography (POCUS) device across the study group, and run the study for a year before collating data and publishing findings.

All enrolled practitioners will undergo comparable training to reduce clinical variance in assessment and interpretation.

My hypothesis is that we will show a significant opportunity in relation to healthcare costs in reducing admissions and iatrogenic harm associated with admission to emergency departments and subsequent wards, where discharge is not easy to arrange, such as with care home residents or house-bound patients. Clinical findings supporting the presence of raised intracranial pressure will also enable the pre-hospital clinician to provide objective evidence of the need for admission where indicated, potentially saving lives as well.

If successful, I will seek support to update NICE guidelines in relation to best practice and move forwards in supporting the adoption of such a technical skill to a wider healthcare community.

## Dragon’s den

### ‘Misfortune Avenue’: bridging the gap between theory and practice in paramedic education using Thinglink as a virtual learning platform

**James Wilkinson**

University of Hertfordshire ORCID iD: https://orcid.org/0009-0001-9959-4632 Email: j.wilkinson3@herts.ac.uk

#### Abstract

**Introduction:** ‘Thinglink’ is a virtual learning platform, allowing users to upload photos and videos to create realistic online worlds. ‘Misfortune Avenue’ is a virtual street, where paramedic students can meet patients from clinical practice as case studies in a safe, online environment. Academic staff can support the learning of theory content from a live teaching session by mimicking real-world environments and can help design management plans to re-enact in simulation. Scenarios are designed to become more complex over three stages, with patient presentations constructively aligned against indicative content from each academic level of a paramedic science degree. The design uses scaffolding of the curriculum, allowing students to build an understanding of how an illness might progress through the longitudinal patient journey, created around various patient presentations commonly seen in paramedic practice. The scope of the platform allows interprofessional working between healthcare workers across the wider health service and encourages learning throughout the continuation of the patient journey.

**Methods**: Phenomenography will explore how students use the virtual environment in their own ways, and focus groups will collect data from first- and second-year paramedic students to better understand whether Thinglink is useful in bridging the gap between theory and practice. The study aims to explore whether ‘Misfortune Avenue’ is beneficial to bridge this knowledge gap as part of the overall learning experience of students using this platform. The objectives are to understand how students are using the technology, if at all, what they are using it for and how they feel the platform can be improved for future use within the curriculum.

**Results:** The findings will be beneficial to paramedic students and healthcare educators to inform the future development of virtual learning resources and encourage consistency across paramedic higher education programmes. The outcome of the research will create a positive impact on student experience and encourage new ways of bridging theoretical knowledge into real-world application outside of placement and simulation.

**Conclusions:** Finding new ways to encourage learning through simulation is part of increasing contact hours through the NHS long-term workforce plan. Simulation also aims to mitigate areas of practice where presentations are less commonly seen and to enhance clinical decision making in a safe environment. The opportunities to augment this with virtual reality are also viable in future research, to create a more high-fidelity and immersive experience.

## Dragon’s den

### Does a student paramedic’s uniform impact their feeling of belonging?

**Adonia McKellow**

University of Sunderland ORCID iD: https://orcid.org/0009-0003-6168-4661 Email: adoniaparamedicscience@gmail.com

#### Abstract

**Introduction:** Before my first clinical placement at university, I was excited about wearing a green uniform in the future; however, I was relieved that I would stand out as a student so that my clinical skills would not be assumed to be higher than they were. After my final placement of year one, I felt very different. I felt part of the team with my mentor and CCA but, simultaneously, excluded due to me standing out as a student and because of the assumption that I would ‘stand and observe’. The aim of this study is to explore whether a student’s uniform influences their sense of belonging, inclusivity and well-being, whether it affects their clinical competence and, finally, whether it impacts their confidence.

**Methods:** It is proposed that a Qualtrics survey is employed ‘nationwide’ using social channels to student paramedics to gauge their opinion on their organisation’s uniform. This would include open/closed questions, gathering information on well-being, confidence and opinion. It is then proposed that a simulation is set up using the University of Sunderland PCPIs, whereby two assessments are undertaken, the first by a student in a traditional uniform and the second by a student in university uniform. The PCPIs will then be interviewed or complete a Qualtrics survey to gather information on whether they have subconsciously/consciously noticed any impact that the uniform has. Finally, it is proposed that a group of students complete a clinical placement in an alternative uniform and following this are interviewed to see if they have noticed an impact on their sense of belonging, inclusivity, confidence and clinical competence. There is, however, an ethical consideration in terms of which university year to choose and how it may affect their career/well-being.

**Results: **Students should feel included with the ambulance staff that they are working with; however, I recognise the importance of it being obvious that they are a ‘student’ and not acting outside their remit. The results of this study could be invaluable for the progression of students through their university course.

**Conclusions:** This study could influence the uniform that universities issue to their students and could aid students’ progression on their career path. There is a consideration that the study may need to be split in order to make the results achievable. Future research may be undertaken comparing university-issued uniforms against each other.

## Dragon’s den

### Perceptions of clinical competence among UK paramedics: a survey-based exploration

**Kate Snowdon**

University of Sunderland Email: kate.snowdon@sunderland.ac.uk

#### Abstract

**Introduction:** Clinical competence is the cornerstone of effective pre-hospital care. While formal standards and guidelines outline the requisite skills and knowledge, the subjective understanding of clinical competence among paramedics, and its perceptual variation among colleagues, remains less explored. This study aims to investigate the concepts that contribute to the formation of clinical competence among UK paramedics.

**Methods:** This study will utilise a mixed-methods design. A Qualtrics questionnaire will incorporate both qualitative and quantitative responses to questions designed to explore paramedics’ perceptions of clinical competence among their colleagues. This will include asking participants to name qualities, attributes and skills that form competence and then rank them in order of importance. Additionally, demographic information, such as years of experience, level of training and current employment role, will be collected.

**Results:** Descriptive statistics will be used to summarise paramedics’ perceptions of clinical competence. Qualitative analysis will be conducted on open-ended responses to identify themes and patterns in the participants’ perceptions. The findings of this study will provide valuable insights into how UK paramedics conceptualise clinical competence within their professional context. This understanding has implications for paramedic education, training methodologies, policy formulation, recruitment strategies and staff retention efforts. Moreover, the data gathered will provide key information on the factors that influence peer or mentor assessment of ability to perform and thrive in the role. The findings could have consequences for the training and education of future paramedics. There are also potential consequences for the resilience and well-being management of paramedics. If the results uncover a particular trend in traits and qualities that are considered to align with clinical competence, this could have both positive and negative influences on individuals who do, or do not, display such qualities and traits.

**Conclusions:** This study will contribute to the existing literature on pre-hospital care by exploring the subjective perceptions of clinical competence among UK paramedics. By examining how paramedics evaluate their colleagues’ clinical competence, this research aims to inform strategies for improving training, promoting professionalism and, ultimately, enhancing patient outcomes in pre-hospital settings.

## Paramedic-performed anaesthesia: a question to be put to sleep? Advanced paramedics’ first-pass success during pre-hospital emergency anaesthesia

**Tom Inkson-McNally**

University of West London Email: tominksonmcnally@gmail.com

### Abstract

**Introduction:** Pre-hospital emergency anaesthesia is the technique of choice for securing the airway of critically ill patients when advanced airway management alone is insufficient. Paramedic-performed pre-hospital emergency anaesthesia is a controversial topic, with previous primary research and systematic reviews concluding poor success rates in comparison with doctor-performed anaesthesia, in addition to high incidents of adverse events. However, more recent primary research has shown that advanced paramedics with post-graduate education equal the first-pass success rates of their doctor counterparts. Thus, a research gap exists. This rapid systematic review looks to close this gap by providing the first review evidence focused solely on advanced paramedics’ first-pass success rates during pre-hospital emergency anaesthesia, aiming to present an evidence base and recommendations for paramedic practice in the UK.

**Methods:** A systemic search of CINHAL Complete, MEDLINE and PUBMED was conducted using PRISMA methodology to identify articles published between 2000 and 2023 on the subject of advanced paramedic pre-hospital emergency anaesthesia. Overall first-pass success, ‘trauma only’ first-pass success and overall success were estimated using a random effects meta-analysis. A narrative synthesis of identified key themes was also conducted.

**Results:** In total, 651 articles were identified and of those seven met the inclusion criteria. Crude means reported overall first-pass success, ‘trauma only’ first-pass success and overall success as 88.5%, 90.4% and 99.2%, respectively. Random effects meta-analysis estimated overall first-pass success, ‘trauma only’ first-pass success and overall success as 89.7% (87.7%–91.8%), 89.6% (86.8%–92.4%) and 98.7% (97.7%–99.7%), respectively. Four identified themes were explored: appropriately trained/educated advanced paramedics are safe at conducting pre-hospital emergency anaesthesia and have comparable first-pass success rates to doctors; high exposure leads to success; clinical governance; and multidisciplinary teams – doctors appear to be the airway operator in more difficult cases.

**Conclusions:** The pooled means generated by meta-analysis show that advanced paramedics conduct pre-hospital emergency anaesthesia to a high standard and have comparable first-pass success rates to their pre- and in-hospital doctor counterparts. This data provides the first step in assessing exclusively advanced paramedics’ performance of pre-hospital emergency anaesthesia. Further research and facilitating actions must be assessed and completed before advanced paramedic pre-hospital emergency anaesthesia can be adopted into practice in the UK.

## Wales Health Drone Research & Innovation Partnership

**Nigel Rees**

Welsh Ambulance Services NHS Trust; Warwick University Medical School Clinical Trials Unit ORCID iD: https://orcid.org/0000-0001-8799-5335 Email: nigel.rees5@wales.nhs.uk

### Abstract

**Introduction: **The use of drones holds enormous potential for healthcare systems, due to their ability to carry products, cover large distances across remote areas, avoid road congestion and reduce carbon emissions. Drones are being used across the world for tasks ranging from the delivery of samples to the deployment of defibrillators to cardiac arrest. However, many clinical, operational and regulatory barriers exist, requiring multidisciplinary collaboration among clinicians, industry, regulators, engineers, leaders and researchers.

**Methods:** The Wales Health Drone Research & Innovation (R&I) partnership is led by the Welsh Ambulance Services NHS Trust (WAST), along with a range of aerospace engineers, industry, health and research partners. This partnership has resulted in many completed and ongoing projects, funded by bodies such as the UK Space Agency, the Welsh Government, the National Institute for Health and Care Research, Health and Care Research Wales and Resuscitation Council UK.

**Results: **We present the following R&I drone use cases from the Wales Health Drone Research & Innovation Partnership:

- Drone delivery of a defibrillator, lowered by winch to a simulated cardiac arrest.
- A fixed-wing Penguin B drone equipped with satellite-enabled technology successfully conducting end-to-end ‘beyond visual line of sight’ flight delivery of a defibrillator by parachute drop to a simulated cardiac arrest.
- A foundation study for drone-based delivery services to support the Welsh NHS: exploration of a medical drone delivery service between the Welsh Blood Service in South Wales and Wrexham to support and potentially replace current carbon-based transport.

**Conclusions: **The Wales Health Drone Research & Innovation Partnership continues to test and develop drone technology, build the body of knowledge, raise public awareness and provide the assurances required for drones to contribute to efficient, effective and sustainable healthcare.

## Deciphering the ambulance clinician’s dilemma: assessing head injuries in older adults across the UK

**Jack Barrett**

South East Coast Ambulance Service ORCID iD: https://orcid.org/0000-0002-0040-537X Email: jack.barrett@secamb.nhs.uk

**Peter Eaton-Williams**

South East Coast Ambulance Service https://orcid.org/0000-0001-5664-3329

### Abstract

**Introduction:** UK ambulance services employ diverse models of care, resulting in 40–60% emergency department (ED) conveyance rates (Nicholson et al., 2022). Head injury conveyance rates for older adults (60+) remain high (60–70%), despite most being mild (Barrett et al., 2023). We aimed to explore ambulance clinicians’ perceptions, experiences and decision-making processes when assessing older adults with head injuries, considering the various factors influencing their clinical decisions.

**Methods:** This study used a mixed-methods sequential explanatory design, comprising an online survey and one-to-one interviews with patient-facing ambulance clinicians in the UK. The survey, distributed through nine ambulance services and via social media, gathered data on clinicians’ experiences of, confidence levels with and perceptions about assessing older adults with head injuries. It focused on exposure frequency, confidence in assessing asymptomatic patients, perceived risks of medications and confidence in the available decision tools. The subsequent interviews delved deeper into the survey responses.

**Results:** We recruited 385 participants, predominantly male paramedics (62%), with a median age of 35 and eight years of ambulance service experience. Participants reported frequent encounters with older adults with head injuries and expressed high confidence in assessing visible injuries, but lower confidence in neurological examinations. Participants found NICE and JRCALC guidelines satisfactory and reported confidence in conveying patients to the ED, but less confidence in alternative referrals or discharges. The interviews revealed two over-arching themes: guideline-based care and patient-centred care, with sub-themes emphasising the importance of shared decision-making, collaboration with other healthcare professionals and safety-netting strategies.

**Conclusion:** While clinicians express confidence in using clinical guidelines for ED conveyances, they often find guidelines overly prescriptive and struggle to translate them for individual cases. There is a need for more patient-centred, holistic decision making, especially considering the unique aspects of head injuries in older adults. Challenges include fear of poor outcomes, limited feedback on patient outcomes and lower confidence in making referral or discharge decisions. Specific guidelines tailored to this demographic, as well as improved support services, may aid in reducing unnecessary ED conveyances.

ReferencesBarrettJ. W.WilliamsJ.SkeneS. S., et al. (2023). Head injury in older adults presenting to the ambulance service: Who do we convey to the emergency department, and what clinical variables are associated with an intracranial bleed? A retrospective case–control study. *Scandinavian Journal of Trauma, Resuscitation and Emergency Medicine*, 31(1), 65. https://doi.org/10.1186/s13049-023-01138-1.37908011
10.1186/s13049-023-01138-1PMC10619243NicholsonH.VossS.BlackS., et al. (2022). Factors influencing conveyance of older adults with minor head injury by paramedics to the emergency department: A multiple methods study. *BMC Emergency Medicine*, 22(1), 184. https://doi.org/10.1186/s12873-022-00747-w.36418963
10.1186/s12873-022-00747-wPMC9682699

## Are paramedics trained to manage maternal cardiac arrest? What can we learn from the experiences of paramedics managing antenatal maternal cardiac arrest in the out-of-hospital environment to guide training?

**Charlotte Dennis**

Queen Mary University of London Email: cejdennis@gmail.com

### Abstract

**Introduction:** There is limited research on out-of-hospital antenatal maternal cardiac arrest. Research in this field is linked to causation of arrest and maternal survival, with little detail of those providing the resuscitation. This research explores the experiences of paramedics required to manage these cases in the out-of-hospital environment and aims to determine whether paramedics feel they are adequately prepared to manage maternal cardiac arrest.

This study aims to understand whether the experiences that paramedics have in managing antenatal maternal cardiac arrest in the out-of-hospital environment can help guide training.

There are three main objectives:

1. To explore the participants’ experiences of attending and managing out-of-hospital antenatal maternal cardiac arrest.
2. To explore the decision-making process in the management of antenatal maternal arrest in the out-of-hospital environment.
3. To identify areas in which training could be altered to improve future patient care from participants’ experiences in managing out-of-hospital antenatal maternal cardiac arrest.

**Methods:** This is primary research, undertaking and analysing six semi-structured interviews with paramedics who had attended an antenatal maternal cardiac arrest within the previous 24 months. Interviews were transcribed and coded in QSR International NVivo version 12, using an inductive thematic approach for analysis.

**Results:** The experiences of paramedics managing antenatal maternal cardiac arrest varied. Challenges specific to the out-of-hospital environment caused delays in patient care, which resulted in additional stress to clinicians. Participants spoke universally that they felt additional training was required to feel confident in managing the patient group and reported that initial training was inadequate, with reports from advanced paramedics noting that uterine displacement either was not performed or was ineffective due to poor technique. Variation occurred in the decision-making process surrounding the performance of an on-scene resuscitative hysterotomy.

**Conclusions:** Heterogeneity in training, interventions and emotional responses to the management of antenatal maternal cardiac arrest support the need for further training, specifically using high-fidelity simulation. The specific challenges faced emphasise the necessity for additional training of advanced paramedics to perform a perimortem hysterotomy to reduce the delay in performing life-saving intervention.

## Examining the occupational stress experienced by ambulance staff: a mixed-methods stress audit

**Zoe Anchors**

University of the West of England ORCID iD: https://orcid.org/0000-0002-3090-2334 Email: zoe.anchors@uwe.ac.uk

**Helen Nicholson**

University of the West of England

**Lee Moore**

University of Bath

**Rachel Arnold**

University of Bath

**Nicola Walsh**

University of the West of England

**Sarah Black**

South Western Ambulance Service NHS Foundation Trust

**Sarah Voss**

University of the West of England

### Abstract

**Introduction:** Ambulance staff have the highest sickness rates, intention to leave and burnout in the NHS, with work-related stress and anxiety recognised as key causes. There are many known work stressors (e.g. excessive workload, operational demands) that have impacted the health and well-being of ambulance staff; however, there is little evidence as to which of these is having the most negative impact, how that impact occurs and who is most at risk. This study investigated which stressors had the most negative impact on health, well-being, performance and intention to leave, whether these impacts were explained by factors such as cognitive appraisals or mental rest and which ambulance staff were most ‘at risk’ of stress-related problems.

**Methods:** A mixed-methods (explanatory sequential design) stress audit was conducted among ambulance service staff from one NHS trust in Southwest England. An online survey (n = 420) assessed elements of the stress process (i.e. stressors, appraisals and mental rest) and key outcomes (i.e. mental health, well-being, perceived performance and intention to leave). Semi-structured interviews (n = 8) with staff explored the findings of the survey in more depth (e.g. why/how impact occurs). Regression analyses examined the relationships between predictors (e.g. stressors) and outcomes (e.g. well-being), and ANOVAs and t-tests explored differences between groups (e.g. bands) on particular variables. A thematic analysis of qualitative data was conducted to provide explanatory analysis of the survey findings.

**Results:** The findings indicated that higher stress levels related to manager support (i.e. upper management prioritising efficiency over staff needs), workplace demands (e.g. operational) and poor relationships (e.g. with non-peer A&E staff) and constant change had the most negative impact on staff (e.g. well-being, intention to leave). Poor mental rest and threat appraisals (i.e. sustained perceptions of ‘overwhelm’) also strongly predicted negative outcomes (e.g. poorer perceived performance). The accumulation of stress and the triggering of negative emotions were further identified as explanatory factors in the stress process. More experienced staff, such as those in Band 6, those working in mixed (i.e. urban and rural) areas and males were found to be more at risk of deleterious outcomes (e.g. mental ill health).

**Conclusion:** The findings of this audit offer a comprehensive and novel insight into the stress experiences of ambulance staff. Practical recommendations are offered to inform the effective development and delivery of tailored stress management interventions that consider both organisational- and individual-level components.

## Using realist approaches to explain and understand the optimal use of paramedics in primary care

**Georgette Eaton**

University of Oxford; London Ambulance Service NHS Trust ORCID iD: https://orcid.org/0000-0001-9421-2845 Email: georgette.eaton@phc.ox.ac.uk

### Abstract

**Introduction:** In response to the unsustainable workload and workforce crises in primary care, paramedics (with their generalist clinical background acquired from ambulance service experience) are increasingly employed in primary care. However, the specific contribution paramedics can offer to the primary care workforce has not been distinctly outlined.

**Methods:** A realist evaluation was undertaken, consisting of three independent but inter-related research studies:

1. In Phase I, a mixed-methods cross-sectional survey of paramedics in primary care in the UK was conducted to comprehend the existing practices of paramedics within the NHS.
2. Phase II involved an analytic auto-netnography, where online conversations among paramedics in primary care were observed in order to understand paramedics’ perceptions of their role.
3. Phase III consisted of focused observations of paramedics working in primary care in 15 sites across England, Northern Ireland, Scotland and Wales. Sixty interviews were undertaken with paramedics (n = 15), patients (or carers) who had received care from paramedics working in primary care (n = 15), general practitioners who worked with paramedics (n = 15), as well as other professionals employed in primary care, such as nurses, pharmacists and administrative staff (n = 15).

**Results:** The culmination of findings from each phase led to the development of a final programme theory, encompassing three conceptual categories: expectations associated with paramedics in primary care; the transition of paramedics into primary care roles; and the roles and responsibilities of paramedics in primary care. Based on the evidence generated, there are four key recommendations regarding how paramedics work in primary care:

1. A clear strategy for communication of the paramedic’s role in primary care;
2. Developing a comprehensive curriculum framework for paramedics in primary care;
3. The need for an effective transition support structure;
4. Changes to legislation and policy.

**Conclusions:** This research uses a novel approach to present empirical evidence of the role of paramedics in primary care across the UK, and offers insights into factors relating to their deployment, employment and how they fit in within the wider primary care team. On this basis, we have produced a series of practice implementation recommendations, as well as highlighting areas for further research in this area.

## Study of aerosols produced by airway procedures in cardiac arrest

**Ruth M. Fisher**

University of Hertfordshire; Yorkshire Ambulance Service NHS Trust ORCID iD: https://orcid.org/0000-0003-3959-4021 Email: r.fisher4@herts.ac.uk

**Robert Chilcott**

University of Hertfordshire

**Julia Williams**

University of Hertfordshire ORCID iD: https://orcid.org/0000-0003-0796-5465

### Abstract

**Introduction: **During the COVID-19 pandemic, repeated changes were made to healthcare standard operating procedures (SOPs) based on the assumed risk of disease transmission associated with aerosol-generating procedures (AGPs) in cardiac arrest management (Christian & Couper, 2020). However, there is no clear evidence that airway management in a cardiac arrest is an AGP. Therefore, the aim of this study is to establish if paramedics were exposed to excess aerosols during intubation and iGel insertion when performing cardiopulmonary resuscitation (CPR) in an out-of-hospital setting.

**Methods: **Ethical approval was granted by the Yorkshire and the Humber – Leeds West Research Ethics Committee (reference 23/YH/0027; IRAS 304724), who granted a waiver of consent.

Real-time aerosol measurements were conducted during airway management procedures during CPR in out-of-hospital settings between June and September 2023. Recruitment criteria were participants >16 years old, in cardiac arrest, who did not survive. Patient-generated aerosols were measured using two portable particle size-counting instruments via silicone tubing. The end of one tube was placed as close to the patient’s mouth as possible, whereas the other was placed away from the patient to acquire background readings. Research paramedics worked in a supernumerary capacity to avoid interference with patient care delivery.

**Results: **A total of 18 participants were recruited, six of whom had airway insertions recorded.

No significant difference was found between background and patient-generated aerosols (p = 0.2629). There were no significant increases in particle number concentration observed in association with intubation or i-Gel insertion (p = 0.9782 and p = 0.1678, respectively).

**Conclusions: **While further research is required to establish exact aerosol generation by individual procedures in cardiac arrest, the current study does not support the assumption that intubation and i-Gel insertion are aerosol-generating procedures during CPR.

ReferencesChristianM. D. & CouperK. (2020). COVID-19 and the global OHCA crisis: An urgent need for system level solutions. *Resuscitation*, 157, 274–276. https://doi.org/10.1016/j.resuscitation.2020.11.004.33181230
10.1016/j.resuscitation.2020.11.004PMC7834189

## Paramedic perceptions of barriers and facilitators to the use of ambulance service appropriate care referral pathways in Northern Ireland: a qualitative study

**Karl Bloomer**

Northern Ireland Ambulance Service ORCID iD: https://orcid.org/0000-0002-7822-4528

**Jamie Scott**

Ulster University ORCID iD: https://orcid.org/0000-0003-2402-021X

**Rebecca Smyth**

Northern Ireland Social Care Council ORCID iD: https://orcid.org/0009-0003-0832-5088

**Julia Wolfe**

Northern Ireland Ambulance Service ORCID iD: https://orcid.org/0000-0002-3104-8360

### Abstract

**Introduction:** Demand for emergency ambulances is increasing every year. Traditionally, the only option for attending paramedics was to transport patients to the emergency department (ED) (Halter et al., 2011). However, the ED is now generally regarded as just one of a range of options available to paramedics to ensure that people receive the most appropriate care.

Ambulance conveyance rates to ED in Northern Ireland (NI) have only occasionally fallen below 75% of incidents. A study examining a diabetic-specific Northern Ireland Ambulance Service (NIAS) referral pathway showed a much lower referral rate than comparable ambulance services, and data analysis has shown that over 70% of potentially eligible patients experiencing a fall are not receiving a falls referral (Bloomer, 2021; Scott, 2020).

This study aimed to identify what paramedics perceive are the barriers and facilitators to the use of appropriate care pathways (ACPs) in NI.

**Methods:** In this single-centre qualitative study, participants were recruited using convenience sampling. Data were collected through semi-structured interviews until data saturation was reached after 11 interviews. They were recorded, transcribed verbatim and thematically analysed.

**Results:** Six main themes emerged during analysis. The participants discussed their perceptions of the barriers and facilitators of utilising ACPs under the auspices of clinical issues, cultural issues, risk, person-centred practice, inter-professional communication and operational infrastructure.

**Conclusions:** The study provides insight into perceived barriers and facilitators to the use of ACPs. The themes identified are consistent with existing literature that calls for standardised pathways across regions. Future research should investigate the link between the NHS 111 service and ambulance demand. In order to facilitate the complex decision making involved in referral, relevant knowledge and skills should be embedded in paramedic education. Efforts should be made to improve inter-professional communication and awareness of the paramedic scope of practice and knowledge base.

ReferencesBloomerK. (2021). A retrospective cross-sectional analysis of re-contact rates and clinical characteristics in diabetic patients referred by paramedics to a community diabetes service following a hypoglycaemic episode. *British Paramedic Journal*, 6(2), 1–9. https://doi.org/10.29045/14784726.2021.9.6.2.1.10.29045/14784726.2021.9.6.2.1PMC841520734539249HalterM.VernonS.SnooksH., et al. (2011). Complexity of the decision-making process of ambulance staff for assessment and referral of older people who have fallen: A qualitative study. *Emergency Medicine Journal*, 28(1), 44–50. https://doi.org/10.1136/emj.2009.079566.20472704
10.1136/emj.2009.079566ScottJ. (2020). Re-contact rates with a UK ambulance service following paramedic referral to a falls prevention service for those aged ≥ 65 years: A retrospective cohort study. *British Paramedic Journal*, 5(2), 18–25. https://doi.org/10.29045%2F14784726.2020.09.5.2.18.33456387
10.29045/14784726.2020.09.5.2.18PMC7783949

## An updated evaluation of hangings attended by ambulance clinicians in the North East of England

**Gary Shaw**

North East Ambulance Service ORCID iD: https://orcid.org/0000-0001-5279-1412 Email: gary.shaw@neas.nhs.uk

**Lee Thompson**

Newcastle Hospitals; North East Ambulance Service ORCID iD: https://orcid.org/0000-0002-0820-1662

**Graham McClelland**

Northumbria University; North East Ambulance Service; Newcastle University; University of Hertfordshire ORCID iD: https://orcid.org/0000-0002-4502-5821

### Abstract

**Introduction: **Hanging, strangulation or suffocation remains the commonest method of suicide nationally, accounting for 60% of deaths. The North East had the highest rate of deaths by suicide in England in 2022, with 12.8 deaths per 100,000 compared to a national average of 10.5 (Office for National Statistics, 2023). This project aimed to describe patients reported as hanging attended by North East Ambulance Service (NEAS) and evaluate the current situation with reference to previous work in this area (Shaw et al., 2021).

**Methods: **A service evaluation of hangings attended by NEAS between 1 December 2020 and 29 February 2024 was undertaken. Patients were identified from the routine trauma audit. Data were manually extracted from the source clinical record.

**Results: **Across 39 months, a total of 2002 completed, threatened or attempted hangings were identified. Children (age <18 years) accounted for 7% (n = 128) of cases in the current evaluation compared to 4% (n = 25) of cases in the previous evaluation. Data on adults is summarised in Table 1.

**Table 1.** Summary data on adults identified as hanging compared with previous evaluation.

	Previous evaluation 1 December 2018 to 30 November 2020	Current evaluation 1 December 2020 to 29 February 2024
Cases	579	1854
Cases per day	0.79	1.56
Median age (years)	35 (27–45)	33 (25–43)
Sex (male)	71%	56%
OHCA	341 (59%)	603 (33%)
OHCA per day	0.47	0.51
Patients with multiple attendances	4	193
Calls to patients with multiple attendances	13	498

The number of cases per day has nearly doubled; the reasons for this may include increased frequency of attempts, the ongoing impact of the pandemic, economic challenges and improved case identification. However, the rate of out-of-hospital cardiac arrest (OHCA) per day has barely changed, indicating that attempted and threatened hangings account for most of the increase. The age profile remains similar; however, the proportions of women and children have both increased. Many callers with multiple attendances were identified, perhaps indicating patients struggling to access the help they need elsewhere.

**Conclusion: **This is one of the largest UK datasets published on this patient group and, when combined with the preceding data, provides a detailed description of 2606 patients across more than five years in the region, with the highest prevalence in the UK. Despite the number of calls resulting in cardiac arrest not increasing greatly, the impact of frequent exposure to these highly traumatic calls needs further investigation. The subset of patients making repeated calls also needs further investigation.

ReferencesOffice for National Statistics. (2023). Suicides in England and Wales: 2022 registrations. https://www.ons.gov.uk/peoplepopulationandcommunity/birthsdeathsandmarriages/deaths/bulletins/suicidesintheunitedkingdom/2022registrations.ShawG.ThompsonL. & McClellandG. (2021). Hangings attended by ambulance clinicians in the North East of England. *British Paramedic Journal*, 6(3), 49–57. https://doi.org/10.29045/14784726.2021.10.29045/14784726.2021.12.6.3.49PMC866963634970082

## Mental well-being of first-year undergraduate student paramedics before and after experience of their first ambulance placement: a mixed-methods pilot study

**Owen Finney**

University of Sunderland ORCID iD: https://orcid.org/0000-0003-3744-2710 Email: owen.finney@sunderland.ac.uk

**Kate Snowdon**

University of Sunderland

### Abstract

**Introduction: **Emergency ambulance workers are at risk of poor mental well-being, with causes being attributed to shift work, stress and exposure to trauma. Student paramedics in the UK conduct ambulance placement as part of their undergraduate training, which exposes them to the same stresses as emergency ambulance workers. There is further evidence to suggest that student paramedics have additional risk factors for poor mental well-being, such as social isolation and transition to adulthood. Despite these increased risks, there is minimal evidence to explore how mental well-being is affected by ambulance placement. This pilot study aimed to describe and explore the mental well-being of UK student paramedics before and after their first ambulance placement.

**Methods: **Mixed methods were used to collect quantitative and qualitative data from a cohort of first-year student paramedics (n = 30) at the University of Sunderland (UoS) before and after their first ambulance placement. Pre- and post-placement questionnaires using the Warwick-Edinburgh Mental Wellbeing Score (WEMBS) and open questions were used. Quantitative data was analysed using descriptive and inferential statistics and the qualitative data was analysed using thematic analysis. Ethical approval was gained from UoS (reference: 015015).

**Results: **Twenty participants were included in the final analysis. During the placement, the participants completed an average of 177.5 hours (SD ±36.5) on ambulance placement, with 75% (15/20) reporting that they had witnessed at least one traumatic event during their placement. Before and after placement WEMBS were summarised. Prior to placement, the group (n = 20) had a pre-placement mean WEMBS of 48.45 (SD ±6.73). Two weeks after placement, the group (n = 20) had a mean WEMBS of 50.55 (SD ±5.9). There was a mild increase of 2.1 in post-placement WEMBS, which was insignificant on statistical testing (p = 0.30). Qualitative thematic analysis identified five themes: positive learning experience; theory–practice gap; student–mentor relationship; student resilience; and career consolidation.

**Conclusions: **This pilot mixed-methods study found that participants’ mental well-being after exposure to their first ambulance placement did not change significantly. Furthermore, thematic analysis of the qualitative results has shown that placement was an overall positive experience. The ambulance placement also confirmed their career choice and allowed the crossing of the theory–practice gap. Students did experience traumatic events, and paramedic mentor support was shown to be an important protective factor. The methodology worked well; however, there were limitations in the size of the sample, which did not meet WEBMS recommendations. Further research should use a similar design on a bigger scale that is conducted to track student well-being over the entirety of their educational programme to identify protecting and damaging factors to well-being.

## Staff and patient insights to improve fatigue management in NHS ambulance services: CATNAPS study

**Chiara Lombardo**

University of East Anglia ORCID iD: https://orcid.org/0000-0002-9705-8094 Email: c.lombardo@uea.ac.uk

**Jennifer Dawe**

University of East Anglia ORCID iD: https://orcid.org/0000-0002-9815-9372

**Elicia Austin**

University of Hertfordshire

**Julia Williams**

University of Hertfordshire

https://orcid.org/0000-0003-0796-5465

**Jeremy Dearling**

PPIRes (Patient and Public Involvement in Research)

**Jonathan Rogers**

East of England Ambulance Service NHS Trust

**Kristy Sanderson**

University of East Anglia; NIHR ARC East of England ORCID iD: https://orcid.org/0000-0002-3132-2745

### Abstract

**Introduction:** Poor sleep and fatigue are common in acute and emergency healthcare staff. CATNAPS is a multi-site, multi-method study that explores how fatigue can be managed in the NHS ambulance workforce.

The overall CATNAPS aim is to develop a comprehensive fatigue risk management system (FRMS) for UK NHS ambulance services, which is acceptable, feasible and likely to improve patient outcomes and staff well-being and experience. This is the first study to develop a comprehensive approach to fatigue management in an NHS workforce.

In this particular sub-study, we investigate how frontline staff and patients experience current fatigue actions and explore the potential to improve safety culture and reporting.

**Methods: **We recruited 30 frontline ambulance staff members and three ambulance service users from two trusts for a one-to-one semi-structured interview. Staff participants were asked about their experience of fatigue, including their engagement with and/or opinions about our co-produced list of components for fatigue management. Patients were also asked for their views about the same recommended actions. All participants were also asked how, and whether, the recommended actions could be implemented, addressing any potential facilitators and barriers.

Thematic analysis was undertaken on the interview data. All members of the research team, including two members from a patient and public involvement perspective, were involved in the analysis.

**Results:** Seven themes explain staff and patient perspectives on fatigue management, including the proposed implementation of the recommended components of the FRMS:

- Fatigue is not spoken about.
- There is a lack of consistency from management.
- Trusts have implemented limited initiatives to manage fatigue.
- Fatigue management is a matter of personal preference.
- There is impact of, and on, the personal lives of staff to consider.
- Staff and patients have views about potential implementation of proposed fatigue management strategies.
- There are concerns that fatigue can impact quality of care.

**Conclusions: **Staff expressed reluctance to report fatigue because of fear of consequences, while both staff and patients supported formalised and evidence-based implementation of fatigue management actions by ambulance trusts. Current actions are predominantly staff led, so they are highly individualised, including caffeine use, role switching and sleep banking. Successful implementation by trusts of preferred fatigue management actions will require buy-in from front-line staff, managerial support, improved resourcing and increased public awareness on the importance of managing fatigue. These insights are informing development of implementation guidance so ambulance services can tailor the FRMS to their staff and their setting.

## An exploration of the experiences of disabled paramedics and student paramedics in their clinical and educational settings

**Nova Bridge**

University of Hertfordshire; College of Paramedics Email: akkikko.jb@gmail.com

### Abstract

**Introduction:** There are approximately 14.6 million disabled people in the UK, with only 4.4 million disabled people of working age in employment. It is well documented that disabled people are more likely to experience prejudice and less favourable treatment in all aspects of their lives, including employment, than those who are not disabled. Published studies exploring the experiences of any disabled healthcare professional in employment and/or education, including paramedics, are extremely limited. Therefore, it was necessary to conduct this research to explore the experiences of disabled paramedics/student paramedics on how their disability affects their clinical practice, working environment and/or education.

**Methods:** Five objectives were identified:

1. What are the challenges faced by paramedics/student paramedics in their professional lives?
2. What are the experiences of reasonable adjustments for disabled paramedics/student paramedics?
3. What are the experiences of paramedics/student paramedics of bullying/harassment in the workplace?
4. What support are disabled paramedics/student paramedics offered in the workplace for their disability?
5. What recommendations do participants have to improve their professional lives?

Thematic analysis was rooted in an interpretive ontological philosophy of an anonymous survey with open and closed questions, Likert scales and binary yes/no questions. Questions explored participants’ experiences of being disabled, the culture of work/education, reasonable adjustments, disability-related sickness, visible role models and support networks.

**Results:** The themes in the data describe both a passively and actively hostile environment for disabled paramedics and student paramedics, where the participants stated they experience barriers in many aspects of their professional lives, a lack of positive and visible disabled role models and a lack of effective support networks. These systemic barriers and negative attitudes surrounding disability may also be reinforcing self-stigma within some of the participants. Reports of positive experiences are very limited.

**Conclusions:** The overall experiences of disabled paramedics and student paramedics are not positive. Additional training, focused on inclusive practices and how to challenge both individual and cultural biases, should be undertaken. Further study should be performed to gather examples of effective practice in reasonable adjustments, as well as inclusive/supportive cultures.

## Paramedic independent prescribing in emergency and urgent care: understanding the benefits, limitations, facilitators and barriers

**Adam Bedson**

South Western Ambulance Service ORCID iD: https://orcid.org/0000-0003-3583-645X Email: adam.bedson@swast.nhs.uk

**Joanne Turnbull**

University of Southampton

**Susan Latter**

University of Southampton

### Abstract

**Introduction:** This research aims to investigate any benefits and limitations of paramedic independent prescribing (PIP) in emergency and urgent care (EUC), exploring facilitators or barriers that might influence implementation and delivery. Currently, a very limited evidence base exists regarding PIP, so it is unclear if and how it is benefitting patients in EUC services, which include emergency departments, urgent care and ambulance services.

**Methods:** A mixed-methods design is being used, which includes a systematic review, semi-structured interviews with subject experts and case study research in an emergency department and urgent care service.

**Results:** The systematic review and subject expert interviews demonstrated how PIP can improve patient care and enhance the contribution of paramedics within EUC services. Key facilitators included sufficient access to patient records, diagnostic test results and medical support. Limitations included the current legislative restrictions preventing paramedics from prescribing controlled drugs, which are frequently required in EUC. It is also unclear if PIP is beneficial in ambulance services, where alternatives for medicine supply may be sufficient and more appropriate.

**Conclusions:** While the introduction of PIP has the potential to improve patient care and NHS service delivery, a range of potential challenges and limitations exist, and these will be explored further during the planned case study research.

## Emergency medical services streaming-enabled evaluation in trauma: the SEE-IT trial

**Lucie Ollis**

University of Surrey ORCID iD: https://orcid.org/0000-0002-8774-7865 Email: l.ollis@surrey.ac.uk

**Cath Taylor**

University of Surrey ORCID iD: https://orcid.org/0000-0001-6239-4744

**Richard Lyon**

University of Surrey; Surrey and Sussex Air Ambulance

**Julia Williams**

South East Coast Ambulance Service NHS Foundation Trust; University of Hertfordshire School of Health and Social Work

**Scott Munro**

University of Surrey; Surrey and Sussex Air Ambulance

**Carin Magnusson**

University of Surrey

**Jill Maben**

University of Surrey

### Abstract

**Introduction:** Video live-streaming from the scene of an incident to emergency medical services (EMS) is becoming increasingly common to aid decision making about resources required. Critical care and helicopter emergency medical services (HEMS) resources are finite, meaning appropriate allocation is vital. Possible benefits of live-streaming include earlier, more appropriate dispatch and associated clinical/financial gains, but current evidence is sparse and methodologically limited. There is a lack of evidence regarding the experience and impact on users (999 callers and those viewing the footage). The aim of this feasibility randomised controlled trial (RCT) was to investigate the feasibility of conducting a definitive RCT of the use of live-streaming for trauma incidents. This presentation will focus on caller and staff experiences.

**Methods:** Feasibility RCT with an embedded process evaluation (surveys, interviews and observations) (Ollis et al., 2023) and two observational sub-studies. During six trial weeks, working shifts in one emergency operations centre (EOC) were randomised 1:1 to live-streaming during eligible trauma incidents (using GoodSAM Instant-On-Scene (GoodSAM, 2023)) or standard care. Progression criteria included:

1. Over 70% of callers with smartphones agreeing and able to activate live-streaming;
2. Over 50% of requests to activate live-streaming resulting in footage being viewed;
3. The HEMS stand-down rate reducing by ≥10% due to live-streaming;
4. No evidence of psychological harm in callers or staff.

**Results:** Sixty-two shifts were randomised, including 240 incidents (132 control; 108 intervention). Live-streaming was successful for 53 incidents. Patient recruitment (to determine appropriateness of dispatch) and caller recruitment (to measure potential harm) were low (58/269, 22% of patients; 4/244, 2% of callers). Two of the progression criteria were met: 86% of callers with smartphones agreed and were able to activate live-streaming; and 85% of requests to activate live-streaming resulted in footage being obtained. Two were indeterminate due to insufficient data: 2/6 (33%) HEMS stand down was achieved due to live-streaming; there was no evidence of psychological harm from the survey, observations or interviews, but there was insufficient survey data from callers or the comparison site to provide confidence. Results suggest live-streaming is feasible to implement, easy to use, acceptable to both lay callers and dispatchers and may aid dispatch decision making. Limitations included the low recruitment of lay public 999 callers, patients and staff from the comparison site (for staff well-being survey).

**Conclusion:** Published findings (Taylor et al., 2024) support the decision to progress to a definitive RCT. Further assessment of unintended consequences, benefits and harms of live-streaming is required.

ReferencesGoodSAM. (2023). *Instant help: Ambulance/Police/Fire*. https://www.goodsamapp.org/instantOnScene.OllisL.SkeneS. S.WilliamsJ., et al. (2023). The SEE-IT trial: Study protocol for an interventional feasibility randomised controlled trial. *BMJ Open*, 13(4), e072877. https://doi.org/10.1136/bmjopen-2023-072877.10.1136/bmjopen-2023-072877PMC1015183437094896TaylorC.OllisL.
LyonR. M., et al. (2024). The SEE-IT trial: Emergency medical services streaming enabled evaluation in trauma: A feasibility randomised controlled trial. *Scandinavian Journal of Trauma, Resuscitation and Emergency Medicine*, 32, 7. https://doi.org/10.1186/s13049-024-01179-0.38383402
10.1186/s13049-024-01179-0PMC10883301

## The effect of feedback on the transition experiences of newly qualified paramedics

**Peter Phillips**

Bournemouth University ORCID iD: https://orcid.org/0000-0002-9895-6945 Email: pphillips@bournemouth.ac.uk

**Steve Trenoweth**

Bournemouth University ORCID iD: https://orcid.org/0000-0001-8342-499X

### Abstract

**Introduction:** Research into the transition experiences of newly qualified paramedics (NQPs) suggests that it can be an emotionally turbulent period (Phillips & Trenoweth, 2023). The social process has been emphasised, where NQPs prioritise fitting in and gaining the acceptance of their colleagues (Devenish et al., 2016). There is evidence that NQPs lack confidence (Health Education England, 2018) and self-efficacy, and that this is a source of anxiety that can cause poor well-being (Phillips & Trenoweth, 2023). This study sought to understand the transition experiences of NQPs in the UK and the effect that these experiences had on their well-being. It presents qualitative findings of a larger study.

**Methods:** A one-year longitudinal design was used with a social constructionist underpinning. Semi-structured interviews were conducted on a convenience sample of 18 NQPs. Constructivist grounded theory was used to conduct and analyse interviews. Initial coding and focused coding were carried out alongside memoing to construct a theory based on participants’ experiences. Relevant ethical approval was granted.

**Results:** The first year of employment in the ambulance service was emotionally turbulent for NQPs. The category ‘Receiving valued and confirmatory feedback’ describes how participants valued clinical feedback from established paramedics. Feedback was often unsolicited and informal. This type of feedback was unlocked and freely offered only when participants had gained membership of the social group. Feedback improved their self-efficacy, as well as further establishing their social cohesion and membership of the established group of paramedics. These factors helped participants to navigate the NQP process. Participants placed less value on formal organisational feedback through the NQP process. For other participants, who did not gain membership of the social group, they did not receive feedback from paramedics and this had a detrimental effect on their self-efficacy and well-being.

These findings support evidence that effective feedback can improve self-efficacy and well-being, and can buffer mental health. Formal feedback mechanisms for paramedics have been found to be lacking, and feedback from colleagues is a source of valued and credible opinion. This study strongly supports emerging evidence that peer-to-peer feedback can support self-efficacy and well-being in paramedics, and this is particularly important for NQPs. 

**Conclusions:** NQPs view informal feedback from paramedics as important and credible. It can improve self-efficacy and well-being. Mechanisms that formalise peer-to-peer feedback may help to make the NQP period less turbulent and improve NQPs’ self-efficacy and well-being.

ReferencesDevenishA. S.ClarkM. J. & FlemmingM. L. (2016). Experiences in becoming a paramedic: The professional socialization of university qualified paramedics. *Creative Education*, 7(6), 786–801. https://doi.org/10.4236/ce.2016.76081.Health Education England. (2018). *Reducing pre-registration attrition and improving retention report*. https://www.hee.nhs.uk/our-work/reducing-pre-registration-attrition-improving-retention.PhillipsP. & TrenowethS. (2023). Crossing the ‘flaky bridge’ – the initial transitory experiences of qualifying as a paramedic: A mixed-methods study. *British Paramedic Journal*, 8(1), 18–27. https://doi.org/10.29045/14784726.2023.6.8.1.18.10.29045/14784726.2023.6.8.1.18PMC1024086137284606

## A review of palliative and end-of-life care education in Scottish paramedic undergraduate courses

**Ross MacInnes**

University of Aberdeen Email: ross.macinnes3@hotmail.co.uk

**Derek Scott**

University of Aberdeen ORCID iD: https://orcid.org/0000-0002-2029-6174

### Abstract

**Introduction: **Paramedics are being increasingly dispatched to community palliative and end-of-life care (EoLC) patients (Kirk et al., 2017). Despite this, paramedics consistently report a lack of confidence in treating these patient populations (Blackmore, 2022). Sufficient education in these areas has been identified as a key enabler to the provision of adequate care for these patients by paramedics (Pentaris & Mehmet, 2019). Scotland has recently moved to a degree-based training programme for paramedics, and it has yet to be assessed whether or not paramedics qualifying in Scotland are being educated adequately in palliative and EoLC topics.

The aim of this study was to review and analyse the palliative and EoLC education within the five Scottish undergraduate paramedic degrees and compare it to the recommendations made by the relevant educational guidance.

**Methods: **Information gathered from publicly accessible online curriculum documents and data collected by contacting course representatives from each university were screened for information regarding the following:

1. Content delivered in modules relating to palliative care and EoLC;
2. Teaching techniques used to deliver palliative care and EoLC teaching content;
3. Number of hours dedicated to the delivery of palliative care and EoLC material.

A thematic analysis was carried out on the data collected regarding course content to gain a comprehensive summary of the educational framework in Scotland. This was compared to the educational recommendations identified.

**Results:** When compared with the educational guidance, five of the identified recommendations were met by two or more universities, three were only met by one university each and one recommendation did not appear to be met by any of the courses studied. The teaching techniques used included lectures, tutorials/workshops, seminars, simulations and self-directed study. The mean teaching time dedicated to this content was 12.9 hours (SD 8.47).

**Conclusion: **Scottish undergraduate paramedic courses largely met the education guidance for palliative and EoLC teaching; however, there is scope for implementing further interactive teaching sessions focusing on these topics to address the gaps in the existing framework and to further develop student confidence in these areas of practice. This report proposes a model for a simulation session in an attempt to address these issues.

ReferencesBlackmoreT. A. (2022). What is the role of paramedics in palliative and end of life care? *Palliative Medicine*, 36(3), 402–404. https://doi.org/10.1177/02692163211073263.35176935
10.1177/02692163211073263PMC8972948KirkA.CromptonP. P.KnightingK., et al. (2017). Paramedics and their role in end-of-life care: Perceptions and confidence. *Journal of Paramedic Practice*, 9(2), 71–79. https://doi.org/10.12968/jpar.2017.9.2.71.PentarisP. & MehmetN. (2019). Attitudes and perceptions of paramedics about end-of-life care: A literature review. *Journal of Paramedic Practice*, 11(5), 206–215. https://doi.org/10.12968/jpar.2019.11.5.206.

## A qualitative exploration of emergency clinicians’ experiences of caring for patients experiencing back pain

**Matt Capsey**

University of Cumbria ORCID iD: https://orcid.org/0000-0003-3659-5344

**Cormac Ryan**

Teesside University ORCID iD: https://orcid.org/0000-0001-5864-4325

**Jagjit Mankelow**

Teesside University ORCID iD: https://orcid.org/0000-0001-9584-991X

**Denis Martin**

Teesside University

### Abstract

**Introduction:** Patients who present to emergency care experiencing back pain have a wider range of underlying causes, including higher rates of serious pathology. Clinical guidelines tend to focus on primary care, citing low rates of serious pathology and advocating conservative management of chronic non-specific back pain. These guidelines appear difficult to apply to emergency care, but little is known about emergency clinicians’ experiences of delivering care to this patient group.

The primary aim of this study was to explore the experiences of emergency clinicians in caring for patients experiencing back pain. Objectives of the study were to explore:

- The understanding of the term ‘back pain’;
- Perceptions of whether these patients make up a significant proportion of the clinicians’ case load;
- The care that clinicians provide and their confidence in offering that care;
- Opinions of what, if anything, would improve care for this patient group.

**Methods:** The study is a qualitative exploration using reflexive thematic analysis to construct themes from a series of semi-structured interviews. The interviewer, and primary coder, is an experienced academic paramedic with an insider position in relation to emergency care. Other members of the research team provided oversight and review.

**Results:** Thirteen interviews were conducted with a range of emergency clinicians (doctors, paramedics, nurses and physiotherapists). Four themes and ten sub-themes were constructed. The four themes were: understanding back pain; emergency medical services as a legitimate choice for patients; benign or sinister pain; and treatment options.

**Conclusions:** Emergency clinicians have a nuanced understanding of back pain and its presentations. Their understanding of back pain recognised it as a complex presentation, but their focus is on identifying serious causes. Participants identified limited formal education on back pain and this gap was plugged by their own and others’ experiences, often through stories of notable episodes. Clinicians were sympathetic to patients experiencing back pain. Patients were perceived as accessing emergency care due to being distressed; clinicians valued the opportunity to take time with patients, but viewed themselves as the ‘back-stop’ in healthcare. When differentiating between benign and sinister presentations it was recognised that serious pathologies, while less common, did present to emergency care, and once red flags had been used to rule in or out serious pathology, other cases could be referred elsewhere. While participants felt supported in offering treatment, many of the treatments used are not advocated in national guidelines.

## Specialist paramedic deployment improves delivery of post-resuscitation care following out-of-hospital cardiac arrest: a retrospective cohort study

**Alan Cowley**

South East Coast Ambulance Service ORCID iD: https://orcid.org/0000-0002-3093-4395 Email: alan.cowley@secamb.nhs.uk

**Paul Rees**

Barts Heart Centre; The Blizard Institute; The Academic Department of Military Medicine ORCID iD: https://orcid.org/0000-0002-6560-6332

**Dan Cody**

South East Coast Ambulance Service

**Eleanor Jaquet**

South East Coast Ambulance Service

**Sophie Clark**

South East Coast Ambulance Service

### Abstract

**Introduction:** The return of spontaneous circulation (ROSC) care bundle is a set of six interventions designed by NHS England (NHSE) for use in the post-resuscitation care of patients who have experienced out-of-hospital cardiac arrest (OHCA). Compliance with these standards is critical in providing optimal, nationally standardised care and in improving patient outcomes. This study aimed to investigate the impact of critical care paramedics (CCPs) on delivery of post-ROSC care.

**Methods:** A retrospective observational study was conducted across a large semi-urban UK ambulance service. All patients with sustained pre-hospital ROSC following resuscitation for OHCA between September 2021 and August 2022 were included. Two groups were compared with regard to post-ROSC care delivery: a standard care group, and a group where additional expertise was provided by deployment of a CCP to the scene.

**Results:** A total of 997 incidents were included in the study, with 891 incidents in the CCP group and 106 incidents in the non-CCP group. The presence of a CCP was associated with a statistically significant increase in compliance with the ROSC bundle. In total, 75% of incidents with a CCP present were fully compliant with the ROSC bundle, compared with 64% of incidents without a CCP. The mean percentage compliance across the six standards was significantly higher in the CCP group than in the non-CCP group. Secondary outcome analysis showed statistically significant benefits in compliance for several care parameters when a CCP was present.

**Conclusions:** This retrospective study confirms that the presence of a CCP can improve the delivery of post-ROSC care. This highlights the potential benefits of having CCPs as part of the standard pre-hospital care resuscitation team for OHCA. Further research is needed to confirm these findings and to examine the relationship between compliance with the ROSC bundle and patient outcomes.
